# Mother-Infant Bonding and Its Associated Factors Among Mothers in the Postpartum Period, Northwest Ethiopia, 2021

**DOI:** 10.3389/fpsyt.2022.893505

**Published:** 2022-07-13

**Authors:** Habtamu Shimels Hailemeskel, Alemwork Baye Kebede, Metsihet Tariku Fetene, Fentaw Teshome Dagnaw

**Affiliations:** ^1^Department of Pediatrics and Neonatal Nursing, College of Health Sciences, Debre Tabor University, Debre Tabor, Ethiopia; ^2^Dessie Comprehensive Specialized Hospital, Dessie, Ethiopia; ^3^School of Nursing and Midwifery, Jigjiga University, Jigjiga, Ethiopia; ^4^Department of Public Health, College of Health Sciences, Debre Tabor University, Debre Tabor, Ethiopia

**Keywords:** post-partum, mother, infant, bonding, quality of bonding

## Abstract

**Background:**

The emotional bond that a mother senses to her infant is essential to their social, emotional, and cognitive development. Understanding the level of mother-infant bonding plays an imperative role in the excellence of care. However, in Ethiopia, there is a paucity of information about mother-infant bonding in the postpartum period.

**Objective:**

This study aimed to assess the level of mother-infant bonding and its associated factors among mothers in the postpartum period, Debre Tabor Town Northwest Ethiopia, 2021.

**Methods:**

A community-based cross-sectional study was conducted with 422 postpartum mothers. The postpartum Bonding Questionnaire was used to assess mother-infant bonding. The Edinburgh Postnatal Depression Scale was used to assess postnatal depression. The level of marital satisfaction was assessed by using Kansas marital satisfaction scale. Social support was assessed by Oslo social support scale. A simple random sampling technique was applied to select study participants. Simple and multiple linear regression were used to identify potential factors associated with the mother-infant bonding scale. A *P*-value of <0.05 was considered to declare statistical significance.

**Results:**

In this study, out of 420 postpartum mothers,53 (12.6%) had a risk for the quality of mother-infant bond difficulties between mother and an infant; 8.1% of mothers had a risk for rejection and pathological anger; 3.6% of mothers had a risk for infant-focused anxiety and 1.9% of mothers had risk for incipient abuse of an infant. Maternal depression status [adjusted β coefficient (β) = 2.31, 95% CI: (1.98, 2.64)], non-union marital status [β = 15.58, 95% CI: (9.88, 21.27)], being government employee [β = −5.68, 95% CI: (−9.71, −1.64)], having current pregnancy complication [β = −7.28, 95% CI: (−12.27, −2.29)], being non-breastfeeding mother [β = 7.66, 95% CI: (2.94, 12.38)], substance use history [β = −6.55, 95% CI: (−12.80, −0.30)], and social support [β = −2, 95% CI: (−2.49, −1.50)] were statistically significant factors for mother-infant bonding.

**Conclusion:**

Generally, a significant number of mothers had mother-infant bonding difficulties in the postpartum period. Preventing strategies for bonding difficulties focus on social support during pregnancy, screening postpartum mothers for postpartum depression, and special attention to substance users, non-union maternal status, and non-breastfeeding mothers.

## Introduction

Maternal–infant bonding is a maternal-driven process of a passionate tie between a mother and her baby (1, 2). Shreds of evidence showed that this passionate bond begins during the prenatal period and remains all over the child's life (3, 4).

The passionate bond that a mother feels with her infant is the pillar of their social, emotional, and cognitive development (5). Maternal-newborn bonding is crucial to infant development, promotes a mother's effective shift into motherhood, and eases enhancement in the mother's child-rearing skills. The initial mother-infant bonding offers the groundwork for the child's forthcoming adaptation, interactions, and social and developmental milestones (6, 7). Maternal–infant bonding may prolong breastfeeding duration (8, 9). Positive maternal-infant bonding in early infancy predicts good children's social skills, school readiness, and academic success in preschool age (10). This is because the influential role of affectionate nurture in the emotional, social and intellectual development of infants is powerful during a critical time for brain development (11).

Impaired bonding has connected to the formation of bargained psychosocial functioning, debilitated attachment of the child to their mother, and augmented risk for deprived coping abilities future in life (12). Lacking a good early bond, children become less happy, dependent, and less resilient adults (11). Evidence revealed that children who suffered neglect in their early years had increased risks for depression, anxiety disorders, and learning and memory impairments due to less development in the left hemisphere, intensified sensitivity in the limbic system, and reduced growth in the hippocampus, respectively (13).

Bonding disorders have three different manifestations. A mild disorder occurs if the mother experiences delays, ambivalence, or loss of emotional response and expresses dissatisfaction with her feelings toward her infant. A rejection of the infant is if the mother exhibits strong negative feelings about the child, such as regret, dislike, or hatred for his birth. Pathological anger is when the mother experiences anger that is controlled with difficulty or may have the drive to harm or kill the child or may lose control at a verbal level and shout and scream at the baby (12).

Different studies revealed that a significant number of women experienced severe bonding disorders, particularly among women with mental health disorders (14–17). The maternal-infant bonding level can be affected by different factors, such as child planning (17), parity (17), social support (15, 17, 18), stress (16), depression (4, 19, 20), history of emotional abuse in childhood, self and family history psychiatric admission and antenatal anxiety (21), and advanced age (22). Additionally, culture could affect the strength of mother-infant bonding (15).

The development of the mother-infant bond is a major focus of obstetric, neonatal, and pediatric care. There is a little shred of evidence regarding mother-infant bonding in Ethiopia, similarly in Africa. Understanding the level of mother-infant bonding and its associated factors during the postpartum period plays an important role in the improvement of postnatal care and contributes significantly to preventing possible mother-infant bonding impairment to safeguarding the wellbeing of mothers and their babies (23) because psychological interventions increase mother-infant bonding strength (5).

This study aimed to assess the level of mother-infant bonding and its associated factors among mothers in the postpartum period in Northwest Ethiopia. This research will add to the scientific community's understanding of the factors that influence mother-infant bonding difficulties. Additionally, this study will be important to stakeholders and policymakers that work on the future health, development, and survival of the infant and mother on local and regional levels to identify and care for mothers with a heightened possibility of bonding difficulties.

## Materials and Methods

### Study Design and Setting

A community-based cross-sectional study was employed in Debre Tabor Town, Northwest Ethiopia. Debre Tabor Town is the capital town of the South Gondar administration zone which is located 667 km away from Addis Ababa, the capital city of Ethiopia, and 103 km from Bahir Dar, the capital city of Amhara regional state. According to the 2015 population census report, the total population of the Zone is around 2,578,906. The place of recruitment for this study was Debre Tabor town which has 6 kebeles (the lowest administrative unit in Ethiopia containing at at-least 5,000 residents). The study was conducted from September 03, 2021–October 15, 2021.

### Study Participants and Eligibility Criteria

All post-partum mothers in Debre Tabor Town were the source population. Postpartum mothers aged older than 18 years and who were up to 42 days postpartum period were recruited for this study. Post-natal mothers who were critically ill and unable to respond or speak and hear during the time of the study period were excluded from the study.

### Sample Size Determination

The sample size for this study was calculated using a single population proportion formula by considering the following assumptions:


$$\begin{array}{l} n\;=\;\frac{\left(Z\frac{\alpha }{2}\right)2\;P\;\left(1\;-\;P\right)}{d2} \end{array}$$

Where:

*n* = minimum sample size required for the study.

Z = standard normal distribution (Z = 1.96) with a confidence interval of 95%.

P (prevalence) = 50% since there is no study conducted in Ethiopia about mother-infant bonding and associated factors.

d = is a tolerable margin of error (d = 0.05), and the sample size was 384. After adding a 10% non-response rate, the overall sample size for this study was 422.

### Sampling Procedure

In the beginning, we checked the number of postpartum mothers from health extension workers to identify eligible postpartum mothers with their corresponding household numbers for all six *kebeles* (the lowest administrative unit in Ethiopia containing at at-least 5,000 residents). Health extension workers have the list of postpartum mothers in each kebeles to facilitate postnatal visits. Using particular household numbers, a list of households containing a coded list of post-partum mothers was ready for each kebeles. Then, proportional allocation to size was done to decide the study participants from each kebeles. Finally, the postpartum mother (0–6 weeks subsequently to birth) was selected using a simple random sampling technique from the listed sampling frame. A house-to-house interview was done based on the particular household numbers for selected postpartum mothers. The lottery method was used to select one participant if there is more than one eligible mother was found in the one selected household. The respondents were considered non-respondents after two revisits were done.

### Study Variables

#### Dependent Variables

Level of mother-infant bonding.

#### Independent Variables

Mother age, mother and husband education, marital status religion, mothers occupation, husband occupation, family income, parity, number of antenatal (ANC) follow up, route of delivery, history of chronic disease, child sex, child age, birth weight, gestational age at birth (term, preterm), child planning, previous pregnancy complication, current pregnancy complication, health condition of the child, breastfeeding, postpartum depression, stress, marital satisfaction, social support, intimate partner violence, and substance use history were independent variables.

### Data Collection Procedures and Measurement

Data were collected using an adapted questionnaire under the guidance of six health extension workers and trained sixteen-degree nurses supervised by other two MSc neonatal nurses. Before having an interview with the mother, data collectors clarified the aim, risks, and possible benefits of the study, the right to refuse to participate in the study, and confidentiality issues.

The postpartum bonding Questionnaire (PBQ) was used to assess mother-infant bonding. The postpartum bonding questionnaire contained 25 questions with 0–125 possible minimum and maximum scores, respectively. The greater the average score of responses reveals more bonding difficulties. PBQ has sub-categorized by four factors. Factor one surveyed the quality of the bond (emotion and affection) between a mother and an infant which contained 12 questions with a maximum score of 60, where the cut point to risk was greater than and equal to 12. Factor two which examined rejection and pathological anger entails 7 questions with a maximum score of 35, where the cut point to be a risk of bonding difficulties was 17. Factor three is about infant-focused anxiety consisting of 4 questions with a maximum score of 20, where the cut point to be the risk of bonding difficulties was 10 and above. Factor four is about incipient abuse of infants and consisted of 2 questions with a maximum score of 10, the cut point to be a risk of bonding difficulties were 3 and more (12). The postpartum bonding Questionnaire (PBQ) validated different samples in the world, including Japan and Belgium. The internal consistency of the questionnaire in the Flemish sample showed a Cronbach's alpha of 0.89 (24, 25). In this study, the reliability coefficient of the postpartum bonding Questionnaire (Cronbach alpha) was 0.83 which indicates good reliability.

Postnatal depression of the mother was assessed by using the Edinburgh Postnatal Depression Scale (EPDS). The EPDS is a 10-item questionnaire, scored from 0 up to 3 (higher score indicating more depressive symptoms), that has been validated for detecting depression in postpartum samples in public health centers in Addis Ababa and showed a sensitivity of 84.6% and specificity of 77.0%. The cut-off for the Ethiopian version of the EPDS was a score of 13 and above for EPDS considered as depressed women and a score below 13 considered as non-depressed women (26, 27).

Intimate partner violence has assessed any physical, psychological, or sexual behavior that harms the mother within a close relationship stated the mother (28). History of substance use was assessed if a women's practice of using any type of substance such as alcohol, tobacco, or cigarette smoking and chat including addictive substances in the current pregnancy.

Marital satisfaction was assessed by using Kansas marital satisfaction scale. Three items Kansas marital satisfaction scale each have a 7-Likert scale ranging from one (extremely dissatisfied) to 7 (extremely satisfied). Total scores range from 3 to 21, with high scores meaning better marital quality (29). Social support was assessed by using Oslo 3-item social support scale. Measures of available support that women perceptive or believe that people in their network will assist in times of need. It consists of three items that ask for the number of close confidants, a sense of concern from other people, and a relationship with neighbors with a focus on the accessibility of practical help. The sum score ranges from 3 to 14, with high values representing strong levels and low values representing poor levels of social support (30).

### Data Quality Control and Analysis

Questionnaires were initially drafted in the English language and then translated into Amharic and retranslated back into English by a language expert. To assure the quality of data training was given to data collectors and supervisors 1 day before and after the pretest about the objective of the study, the tool, ethical issues, and methods of the interview. A pretest was done at Woreta town by using 10% of the total sample size (42 postpartum women) earlier than the actual data collection period to judge the instrument, and amendments were done accordingly. Data were checked for completeness and cleaning was done by visualizing, calculating frequencies, and sorting after exporting SPSS.

The collected data were entered to Epi-data manager version 4.4.1 and exported to statistical package for social science (SPSS) version 25 software for analysis. Multiple linear regression analyses were used to identify the association between the independent and the outcome variable. A scatter plot was used to check the assumption of linearity. Histogram and P-P plots were used to check the assumption of normality. The assumption of independence of errors and autocorrelations was checked by the Durbin Watson statistic (1.7). The equal variance (homoscedasticity) was checked by using the Lvenes test *p*-value which is an objective test. Variance Inflation Factor (VIF) and Tolerance test were used to check multicollinearity. Variables that had a *P*-value of <0.25 in the simple linear regression were retained in a multivariable model to control the possible confounding and a *P*-value of <0.05 was declared as statistically significant.

## Results

There were 543 postpartum mothers in Debre Tabor Town during the study period. A total of 422 mothers were selected from 543 postpartum mothers by using a simple random sampling method. During house-to-house interviews, nine mothers were replaced by others through random methods from the existing frame due to not meeting the inclusion criteria. Out of 422 postpartum mothers, two mothers refused to participate. Finally, 420 mothers were interviewed and analyzed for this study.

### Socio-Demographic Characteristics

The mean age of respondents was 28 (SD = 6.3 years). Most of the participants 365 (86.9%) were Orthodox Christian. Ninety-five percent of the respondents (68.7%) were married. One-fourth of the respondents were unable to read and write 105 (25%). Regarding mothers' occupations, 225 (53.6%) of them were housewives and 65 (15.5%) of them were governmental employees. Twenty-six percent of husbands' educational statuses were college and above (Table 1).

**Table 1:** Socio-demographic characteristics of the post-partum mother in Debre Tabor Town North West Ethiopia 2021.

**Variable**	**Category**	**Frequency**	**Percent**
Marital status	Single	10	2.4
	Married	400	95.2
	Separated	5	1.2
	Windowed	5	1.2
Religion	Orthodox	365	86.9
	Protestant	15	3.6
	Muslim	40	9.5
Mothers educational status	Unable to read and write	105	25.0
	Read and write	100	23.8
	Primary (1–8)	85	20.2
	Secondary (9–12)	60	14.3
	College and above	70	16.7
Husband educational status	Illiterate	105	25.3
	Read and write	70	16.9
	Primary	95	22.9
	Secondary	35	8.4
	College and above	110	26.5
Mothers occupation	Housewife	225	53.6
	Self-employment	65	15.5
	Governmental employee	65	15.5
	Merchant	55	13.1
	Student	10	2.4
Husband occupation	Farmer	165	39.8
	Gov't employee	120	28.9
	Merchant	105	25.3
	Daily laborer	15	3.6
	Others^*^	10	2.4

* *Soldger, unemployed*.

### Maternal Obstetric-Related Characteristics

Most mothers 360 (85.7%) have antenatal care (ANC) follow-up during this pregnancy. Among those who have ANC follow-up, 175 (48.6%) have four times and above ANC follow-up. Concerning the route of delivery, the majority 375 (89.3%) of mothers had a vaginal route of delivery. Fifty mothers (11.9%) encountered complications during this pregnancy. Among those who had a complication during this pregnancy, the premature rupture of membrane (PROM) was the most common complication during this pregnancy 17 (34%). Regarding the place of delivery, 20 mothers (9.2%) had home delivery. Eleven percent of mothers had a history of abortion. Fifty-five (13.1%) mothers encountered Intimate partner violence during this pregnancy (Table 2).

**Table 2:** Maternal obstetric- related characteristics of post-partum mothers in Debre Tabor Town North West Ethiopia 2021.

**Variables**	**Category**	**Frequency**	**Percent**
ANC	Yes	360	85.7
	No	60	14.3
No of ANC	One	10	2.7
	Two	80	22.2
	Three	95	26.4
	Four and above	175	48.6
The current route of delivery	Vaginal route	375	89.3
	Cesarean section	45	10.7
Complications during this Pregnancy	Yes	50	11.9
	No	370	88.1
Type of complication	Urinary tract infection	10	20
	Prolonged/obstructed	12	24
	Premature rupture of membrane	17	34
	Others^*^	11	22
Pregnancy intention	Wanted	385	91.7
	Unwanted	35	8.3
Place of delivery	Heath institution	381	90.7
	Home	39	9.3
Abortion history	Yes	50	11.9
	No	370	88.1
Skin-to-skin contact with their infant	Yes	385	91.7
	No	35	8.3
Intimate partner violence	Yes	55	13.1
	No	365	86.9
Type of violence	Physical	20	36.4
	Psychological	10	18.2
	Psychological and physical	20	36.4
	Sexual	5	9.0

* *Cord prolapse; APH, antepartum hemorrhage; PIH, pregnancy-induced hypertension*.

### Substance Use

Among the respondents, 55 mothers (13.1%) had a substance use history during their current pregnancy. Alcohol was the most common substance (81.8%) among those who had a history of substance use during current pregnancy (Table 3).

**Table 3:** History of substance use in the past 3 months among postpartum mothers in Debre Tabor Town North West Ethiopia 2021.

**Variables**	**Category**	**Frequency**	**Percent**
History substance use in the past 3 months	Yes	55	13.1
	No	365	86.9
Type of substance use	Chat	10	18.2
	Alcohol	45	81.8

### Neonatal Related Characteristics

Most of the neonates (88.1%) were gestational age greater than and equal to 37 weeks (Full-term). More than half of the neonates were male (61.9%). Forty-five (10.7%) neonates were sick during the data collection period. Most of the neonates (91.7%) were fed breast milk exclusively (Table 4).

**Table 4:** Neonatal-related characteristics among post-partum mothers in Debre Tabor town North West Ethiopia 2021.

**Variables**	**Category**	**Frequency**	**Percent**
Gestational age at birth	Greater than and equal to 37 weeks (Full-term)	370	88.1
	Less than 37 weeks (pre-term)	50	11.9
Sex	Male	260	61.9
	Female	160	38.1
Child illness	Yes	45	10.7
	No	375	89.3
Breastfeeding	Yes	385	91.7
	No	35	8.3

### Mothers to Infant Postpartum Bonding

In this study, 53 (12.6%) mothers have a risk for the first factor which is the quality of mother-infant bond (emotion, affection) difficulties between mother and an infant. Thirty-four postpartum mothers (8.1%) have risk rejection and pathological anger (factor-two). Fifteen (3.6%) of mothers had a risk for infant-focused anxiety (factor three). Less than two percent (1.9%) of mothers had incipient abuse of infants (factor-four) (Table 5).

**Table 5:** Mothers to infant postpartum bonding among postpartum mothers in Debre Tabor Town North West Ethiopia 2021.

**Factor**	**Category**	**Frequency**	**Percent**
Factor 1 (mother and infant bond quality)	Risk	53	12.6
	Not risk	367	87.4
Factor 2 (rejection and pathological anger)	Risk	34	8.1
	Not risk	386	91.9
Factor 3 (infant-focused anxiety)	Risk	15	3.6
	Not risk	405	96.4
Factor 4 (incipient abuse of infant)	Risk	8	1.9
	Not risk	412	98.1

### Factors Associated With Mother-Infant Bonding Among Post-natal Mothers

In simple linear regression analysis, age of mothers, number of gravidities, number of parity, number of antenatal care (ANC), marital status, father level of education, mother occupation, sex, mode of delivery, abortion history, chronic disease, current pregnancy complication, skin-to-skin contact, breastfeeding, intimate partner violence, and substance use history were significantly associated mother to infant bonding score. In multiple linear regressions, maternal depression non-union marital status, government employee, current pregnancy complications, non-breast-feeding, substance use, and social support were significantly associated with mother-to-infant bonding difficulties.

Keeping other variables constant, a unit increase in maternal depression score will increase the mother-infant bonding difficulties by 2.31 times [β = 2.31, 95% CI: (1.98, 2.64)]. Non-union marital status mothers were at fifteen times higher risk of mother-infant bonding difficulties than married mothers [β = 15.58, 95% CI: (9.88, 21.27)]. Government employee mothers were five times less at risk of mother-to-child bonding difficulties compared to non-employed mothers [β = −5.68, 95% CI: (−9.71, −1.64)]. Mothers who have no current pregnancy complications were seven times less at risk of mother-to-child bonding difficulties than those mothers who have current pregnancy complications [β = −7.28, 95% CI: (−12.27, −2.29)]. Non-breast-feeding mothers were at seven times higher risk of mother-to-child bonding difficulties compared with breast-feeding mothers [β = 7.66, 95% CI: (2.94, 12.38)]. Mothers who had not a history of substance use were six times less at risk of mother-to-child bonding difficulties than mothers who had a history of substance use [β = −6.55, 95% CI: (−12.80, −0.30)]. A unit that increases the social support score will decrease the mother-infant bonding difficulties by two times [β = 2, 95% CI: (−2.49, −1.50)].

The formula was Mean mother-infant bonding (MIB) Score= 20.3 + (2.31) depression + (15.58) non-union + (7.66) breastfeeding—(5.68) government employee—(7.28) current pregnancy complication—(6.55) substance use history—(2) Social support. The model sufficiency for this study was ANOVA = 22 (16, 303) with *P* < 0.001 and the R-square was 0.55 where 55% of the variability in the Mean MIB Score was explained by the variables associated with it in the final model (Table 6).

**Table 6:** Multivariable distribution of mother-infant bonding among post-natal mothers by key predictors in Debre Tabor Town, Northwest Ethiopia, 2021.

**Variables**	**Category**	**β Coefficients**	***p*-value**	**95% confidence interval of β**
(Constant)	NA	20.3	<0.001	(7.48, 33.21)
Age	NA	−0.24	0.092	(−0.52, 0.04)
Income	NA	0.001	0.44	(0.001, 0.663)
Gravidity	NA	−0.727	0.63	(−3.69, 2.24)
Parity	NA	−0.41	0.75	(−3.36, 2.55)
Number of ANC visit	NA	0.28	0.6	(−0.75, 1.30)
Depression	NA	2.31	<0.001	(1.98, 2.64)^*^^*^^*^
Marital status	Union	Ref.		
	Non-union	15.58	<0.001	(9.88, 21.27)^*^^*^^*^
Father Education	Illiterate and able to read and write	Ref.		
	Primary	−1.46	0.35	(−4.5, 1.5)
	Secondary	−0.84	0.65	(−4.48, 2.81)
	College and above	−0.48	0.83	(−4.89, 3.93)
Mother occupation	Non-employed	Ref.		
	Government employee	−5.68	0.006^*^	(−9.71, −1.64)^*^^*^
Sex	Male	Ref.		
	Female	−1.36	0.32	(−4.08, 1.35)
Route of delivery	Vaginal route	Ref.		
	cesarean section	−0.42	0.87	(−5.39, 4.56)
Current pregnancy complication	Yes	Ref.		
	No	−7.28	0.004^*^	(−12.27, −2.29)^*^^*^
Breastfeeding	Yes	Ref.		
	No	7.66	0.002^*^	(2.94, 12.38)^*^^*^
Intimate partner violence	Yes	Ref.		
	No	−4.77	0.065	(−9.83, 0.29)
Substance use history	Yes	Ref.		
	No	−6.55	0.04^*^	(−12.80, −0.30)^*^
Social support	NA	−2	<0.001^*^	(−2.49, −1.50)^*^^*^^*^

* *Shows p < 0.05 = significant association; NA, Not Applicable*.

## Discussion

This study aimed to assess the level of mother-infant bonding and its associated factors among mothers in the postpartum period, in Debre Tabor town northwest Ethiopia. This study revealed that out of 420 postpartum mothers, 53 (12.6%) mothers have a risk for quality of mother-infant bond difficulties between mother and an infant. Thirty-four postpartum mothers (8.1%) and 15 (3.6%) mothers had a risk of rejection and pathological anger and risk for infant-focused anxiety, respectively. Almost two percent (1.9%) of mothers had incipient abuse of infants. Maternal depression, non-union marital status, government employee, current pregnancy complications, non-breast-feeding, substance use, and social support were significantly associated with mother-to-infant bonding difficulties.

Fifty-three (12.6%) mothers have a risk for the general factor which is the quality of mother-infant bond (factor-1) difficulties between mother and an infant. This is higher compared to a study done in Slovakia that identified that 9.5% of women were at-risk in the Factor-1 (17). Thirty-four (8.1%) of postpartum mothers and 3.6% of postpartum mothers have risk rejection and pathological anger (factor-2) and infant-focused anxiety (factor-3), respectively. This is also higher compared to a study done in Slovakia (17). In this study, <2% (1.9%) of mothers had incipient abuse of infants (factor-4). This was lower compared to a study done in Slovakia which was 5% (17). The difference might be sample size, and study setting including differences in the characteristics of study participants and socio-cultural variation. This implies that particular attention should be given to postpartum mothers' and screening and appropriate prevention strategies for bonding difficulties should be planned in North West Ethiopia.

In this study, maternal depression was significantly associated with maternal-infant bonding difficulties. This is similar to a different study conducted in Norway and Poland where postpartum depression is significantly associated with maternal-infant bonding (16, 19, 20, 31). This may be due to mothers with depressive symptoms displaying less sensitive conduct toward their infant and tending to reply to their children's requirements in a less alert, concentrated, and nurturing way. This implies that screening for postpartum depression has an inevitable role to prevent bonding difficulties. Non-union mothers were at increased risk of maternal-infant-bonding difficulties. This is a similar finding to a study done in Germany (32). This may be due to mothers who are single or widowed or divorced may have greater economic difficulties and may have less time for their children's care resulting from a female-headed household and this may be due to a lack of emotional and social support from their partner, and being unable to share information with their partners during the motherhood transition may increase stress, lower self-esteem, and unhappiness, which lead to bonding difficulties. This implies special attention to non-union mothers and counseling them may alleviate mother-infant bonding.

In our study being a government, employees decreased the risk of mother-to-child bonding difficulties. The study done by the Urban Institute had a similar finding that children who experience an unemployment event in their families are also more likely to see a destabilizing change in family arrangements and decreased family bonding (33). This may be due to mothers who are working in government institutions, which may increase financial resources, which potentially reduces stress, and enhances bonding. Additionally, having no current pregnancy complications has decreased the risk of maternal-infant bonding difficulties. This is similar to other studies (19, 20). This may be due to mothers who have not current pregnancy complications are free from distress and burden; this again decreases mother-infant bonding difficulties. This implies that mothers who have pregnancy complications need attention and psychological reassurance to prevent mother-infant bonding difficulties.

This study showed that non-breastfeeding mothers increased the risk of maternal-child-bonding difficulties. This may be due to breastfeeding forming a bonding involvement between the mother and an infant due to skin-to-skin contact, more rubbing, and holding (17, 22, 34). Maternal-infant touch behaviors, particularly affectionate touch, are influenced by skin-to-skin contact to boost the release of oxytocin. This hormone promotes sociability, reducing fear and anxiety (39). This implies promoting breastfeeding has paramount importance to prevent bonding difficulties. However, this result is different from the study done in Israel in which there was no association between feeding type and bonding (35). This difference may be due to socio-demographical and cultural differences among the study groups. Mothers who have no history of substance use have decreased the mean maternal-child-bonding score. This is in line with other study findings (31). This may be due to substance use having the possibility to damp down the ability of mothers to create an effective connection. Substances may reduce the strength of feeling toward an infant and consequently affect postpartum bonding. Substance use may have an impact on women's emotions and behavior. This implies that screening for substance use and appropriate intervention for the user mothers could decrease bonding difficulties in the postpartum period.

Mothers who have more social support had decreased the mean maternal-child-bonding score. This finding is similar to other study findings (5, 18). This might be due to mothers who had emotional support made happy in their living and making mothers have strong bonding with their children. This implies that those women who believe that no someone will assist in times of need should be linked to social support groups may reduce the occurrence of mother-infant bonding difficulties. In our study, there was no significant difference between pre-term and full-term birth with the risk of maternal-child-bonding difficulties. This finding is with other study findings (36–38).

The strength of this study was since it was community-based and recruited by using probability sampling (simple random), the findings can be inferred to the general population and address those mothers who did not seek care from a health institution. However, this study has some limitations. Because of the cross-sectional nature, it does not show a causal relationship between the variable and outcome. Self-reported method of data collection is exposed to social desirability bias which may result in an overestimation of the level of mother-infant bonding. But this issue was minimized by the use of a similar sex data collector during the interview and probing the respondents was done.

## Conclusion

A high number of mothers had bonding difficulties in the quality of the mother-infant bond, rejection, pathological anger, infant-focused anxiety, including incipient abuse of an infant. Maternal depression status, non-union marital status, being a government employee, having current pregnancy complications, being a non-breastfeeding mother, substance use history, and social support were predictors for mother-infant bonding. Preventing strategies for bonding difficulties focus on social support during pregnancy and screening postpartum mothers for postpartum depression, and special attention to substance users, non-union maternal status, and non-breastfeeding mothers.

## Data Availability Statement

The raw data supporting the conclusions of this article will be made available by the authors, without undue reservation.

## Ethics Statement

The studies involving human participants were reviewed and approved by Debre Tabor University, College of Health Sciences, and Institutional Review Board (IRB). Written informed consent for participation was not required for this study in accordance with the national legislation and the institutional requirements.

## Author Contributions

HH was involved in conceptualization, design, data collection, and analysis in all stages of this article. AK, MF, and FD were involved in the design, analysis, and all stages of this study. All authors read and approved the final manuscript.

## Conflict of Interest

The authors declare that the research was conducted in the absence of any commercial or financial relationships that could be construed as a potential conflict of interest.

## Publisher's Note

All claims expressed in this article are solely those of the authors and do not necessarily represent those of their affiliated organizations, or those of the publisher, the editors and the reviewers. Any product that may be evaluated in this article, or claim that may be made by its manufacturer, is not guaranteed or endorsed by the publisher.

## Abbreviations

EPDS: Edinburgh Postnatal Depression Scale
MIB: Maternal-Infant Bonding
SPSS: Statistical Package for Social Sciences
PBQ: Postpartum Bonding Questionnaire
PROM: Premature Rapture of Membrane
ANC: Antenatal Care.
